# Supplementary material for: Comprehensive infectious disease screening in a cohort of unaccompanied refugee minors in Germany from 2016 to 2017: A cross-sectional study
Source: PLoS Med. 2020 Mar 31;17(3):e1003076. doi: 10.1371/journal.pmed.1003076 (PMC7108686; doi:10.1371/journal.pmed.1003076)
Supplement: S1 Text — The questionnaire was completed by the physician performing the screening visit. (PDF) [file pmed.1003076.s002.pdf]

To:
[Name]
[Address]

[Location], [Date]

Regarding:
[Patient first and FAMILY NAME], born on [Date]
residing at [Address]

To whom it may concern,

We would like to report on the health status of [Patient name], an unaccompanied refugee minor. During our medical appointment on [Date], [Translator's name] provided translation services.

Patient profile

Name*: *(*as registered)*	Date of birth*: *(*as registered)*
Additional names provided (if applicable):	Additional dates of birth provided (if applicable):
Gender:	Arrival in Germany on (Date):
When left country of origin:	Migration route:
Country of origin: [Select] .... or if necessary, enter other:	
In the event of having lived in other/additional countries, then where and for how long:	
Languages: ☐ German ☐ English ☐ French ☐ Italian ☐ Albanian ☐ Russian ☐ Arabic ☐ Kurdish ☐ Farsi ☐ Tigrinya ☐ Wolof ☐ Somali ☐ Fula ☐ Oromo ☐ Krio ☐ Mandinka ☐ Pular ☐ Amharic ☐ Dioula ☐ Jola Additional/other languages:	
Patient's favorite activities, job aspirations:	
**Further patient profile notes**:	

Medical history

	No	Yes
Coughing for over 2 weeks?	☐	☐
Unexplained weight loss?	☐	☐
Chronic illnesses?	☐	☐
Prior operations or accidents?	☐	☐
Allergies?	☐	☐
Sleep disturbances?	☐	☐
Regular medications taken?	☐	☐
Past medical treatments?	☐	☐
Known diseases in family history?	☐	☐
Smoking, alcohol or drug dependency?	☐	☐
Vaccination documentation available?	☐	☐
**Current diseases and/or complaints**:		

Clinical findings

Age*: [ ] years + [ ] months *(*as registered)*			Blood pressure [right/left] upper arm: mmHg			
Weight: kg			Pulse: /min.			
Height: cm			Temperature: °C			
	**normal**	**patho-logical**			**normal**	**patho-logical**
General appearance:	☐	☐		Lungs (incl. respiratory rate):	☐	☐
Nutritional status:	☐	☐		Abdomen:	☐	☐
Skin:	☐	☐		Size of liver and spleen:	☐	☐
— Scabies:	☐	☐		Spine:	☐	☐
Ear, nose & throat:	☐	☐		Extremities:	☐	☐
Eyes:	☐	☐		Neurological status:	☐	☐
Dental status / cavities:	☐	☐		Psychological state (cursory):	☐	☐
Heart:	☐	☐		Other (1): [Description]	☐	☐
Genitals:	☐	☐		Other (2): [Description]	☐	☐
**Further notes on the clinical findings**:						

Medical tests

	**Not conducted**	**Pending**	**Normal**	**Patho-logical**
**Hearing test**:	☐	☐	☐	☐
**Vision**: Vision test:	☐	☐	☐	☐
Color blindness:	☐	☐	☐	☐
Stereo vision:	☐	☐	☐	☐
**X-ray of thorax**: Indications for tuberculosis?	☐	☐	☐	☐
Where: [Name and address of medical facility]]				
**Lab work**: Differential blood film:	☐	☐	☐	☐
• Eosinophilia: /µl	☐	☐	☐	☐
• Anemia:	☐	☐	☐	☐
• Other:	☐	☐	☐	☐
Quantiferon:	☐	☐	☐	☐
HBs-Ag:	☐	☐	☐	☐
HIV serology:	☐	☐	☐	☐
Urine status:	☐	☐	☐	☐
**Other medical tests**: [Description]	☐	☐	☐	☐
**Further notes on the medical tests**:				

Vaccinations

Vaccinations performed at the time of the present appointment:

☐ MMRV ☐ MMR ☐ Varicella ☐ Influenza ☐ dTap IPV ☐ dTap ☐ dT ☐ Other:

Next recommended vaccinations:

☐ MMRV ☐ MMR ☐ Varicella ☐ Influenza ☐ dTap IPV ☐ dTap ☐ dT ☐ Other:

Diagnoses

- [Enter description]

Therapies

- [Enter description]

Summary

In summary, we find the health status of [Patient name] to be: [enter brief text description].

Best regards,

[Name of physician]
