# Supplementary material for: Comprehensive infectious disease screening in a cohort of unaccompanied refugee minors in Germany from 2016 to 2017: A cross-sectional study
Source: PLoS Med. 2020 Mar 31;17(3):e1003076. doi: 10.1371/journal.pmed.1003076 (PMC7108686; doi:10.1371/journal.pmed.1003076)
Supplement: S1 Table — (DOCX) [file pmed.1003076.s006.docx]

**Supplementary Table 1.**

| Country | n |
| --- | --- |
| Gambia | 175 |
| Guinea | 173 |
| Eritrea | 155 |
| Somalia | 119 |
| Ivory Coast | 39 |
| Afghanistan | 29 |
| Morocco | 24 |
| Sierra Leone | 24 |
| Ethiopia | 23 |
| Mali | 13 |
| Senegal | 12 |
| Sudan | 12 |
| Syria | 12 |
| Libya | 11 |
| Algeria | 8 |
| Iraq | 8 |
| Nigeria | 8 |
| Liberia | 6 |
| Guinea-Bissau | 5 |
| Ghana | 4 |
| Cameron | 4 |
| Egypt | 3 |
| Albania | 3 |
| Burkina Faso | 2 |
| Mauretania | 2 |
| Pakistan | 2 |
| Togo | 2 |
| Tunisia | 2 |
| Benin | 1 |
| Gabun | 1 |
| Iran | 1 |
| Jemen | 1 |
| Niger | 1 |
| Serbia | 1 |
| Tschad | 1 |
| Unknown | 3 |
