# Supplementary material for: Comprehensive infectious disease screening in a cohort of unaccompanied refugee minors in Germany from 2016 to 2017: A cross-sectional study
Source: PLoS Med. 2020 Mar 31;17(3):e1003076. doi: 10.1371/journal.pmed.1003076 (PMC7108686; doi:10.1371/journal.pmed.1003076)
Supplement: S2 Table — (DOCX) [file pmed.1003076.s007.docx]

**Supplementary Table 2.**

| Diagnosis | Clinical symptom (% of patients (n)) | | | Mean body mass index (BMI; kg/m^2^) |
| --- | --- | --- | --- | --- |
|  | cough >2 weeks | weight loss | fever |  |
| Negative screening (n=799) | 5.5% (n=44) | 3.4% (n=27) | 1.0% (n=8) | 21.9 |
| Latent tuberculosis (n=38) | 18.4% (n=7) | 10.5% (n=4) | 2.6% (n=1) | 21.0 |
| Active tuberculosis (n=15) | 26.7% (n=4) | 20.0% (n=3) | 0% (n=0) | 20.3 |
