# Supplementary material for: Comprehensive infectious disease screening in a cohort of unaccompanied refugee minors in Germany from 2016 to 2017: A cross-sectional study
Source: PLoS Med. 2020 Mar 31;17(3):e1003076. doi: 10.1371/journal.pmed.1003076 (PMC7108686; doi:10.1371/journal.pmed.1003076)
Supplement: Attachment — Submitted filename: PBP_final.docx [file pmed.1003076.s008.docx]

**Comprehensive infectious disease screening among a cohort of 890 unaccompanied refugee minors in Germany from 2016 to 2017 –**

**Point by point reply**

Dear Reviewers,

We would like to take this opportunity to express our gratitude for the remarkably thorough reviews and highly constructive suggestions. We addressed all questions and are convinced that this significantly improved our manuscript.

Requests from the editors:

Please provide a fuller account of the ethical situation, in which you discuss the responsible party for unaccompanied minors; the issue of informed consent not having been obtained; and the accountability for providing appropriate treatment for the diseases identified.

Thank you for suggesting to give more detailed information on the ethical situation. A legal guardian was assigned for each unaccompanied refugee minor from the local municipality. However, due to the high numbers of refugees during the years 2016-2017, it was logistically not possible for the guardian to be present at the screening visit, since the assigned person was usually legally responsible for a number of refugees. Thus, it was not possible to prospectively obtain informed consent. Many refugees only stayed in our area for a couple of weeks before being distributed to other regions in Germany, therefore contacting the refugees and their legal guardian after the screening visit was also very difficult. We extensively discussed the situation with our ethics committee who granted approval for the study. Importantly, since our electronic questionnaire used by the physicians for the screening (Supplement S1) allowed for direct export of the data into a Microsoft Excel spreadsheet, we were able to collect and analyze the data in a fully anonymized fashion.

With regards to the management of the diseases identified, refugee minors in Germany profit from fully covered health care costs through a governmental health insurance, ensuring appropriate treatment options identical to non-immigrant children in Germany.

We included a clarification on these important ethical issues in the methods section.

**Page 7 Line 208-215**

Please remove "890" from your title, and add a study descriptor to the title following a colon, e.g.
"... : a cross-sectional study".

Thank you for this suggestion. We included the study design changing the title as follows:

*“Comprehensive infectious disease screening among a large cohort of unaccompanied refugee minors in Germany: a cross-sectional study”*

We ask you to combine the "aims" subsection of your abstract with the preceding "background" subsection.

We agree with the editors that a combination of the two subsections makes adds clarity to the abstract and changed it accordingly.

Please combine the "methods" and "findings" subsections of your abstract, and add a new final sentence to the new combined subsection summarizing the study's main limitations.

We appreciate this recommendation and included the most important study limitations which is the potential diagnostic gaps in parasite workup and the unavoidable selection bias in our study cohort. We restructured the abstract and also made small changes towards a sharper conclusion

Please make that "sociodemographic" at line 35, and "January" at line 38.

We corrected those errors.

At line 48, please start the sentence with "In this study, we found that ..." or similar.

We are grateful for this constructive suggestion and changed the conclusion subsection.

After your abstract, we will need to ask you to add a new and accessible "author summary" section in non-identical prose. You may fine it helpful to consult one or two recently published research papers in PLOS medicine to get a sense of the preferred style.

We added this section after the abstract.

**Page 3**

Please remove the text at line 104-107, or move it to the discussion section.

Lines 104-107 were deleted.

Early in the methods section of your main text, please state whether or not the study had a protocol or prespecified analysis plan, and if so attach the relevant document(s) as a supplementary file. Please highlight analyses that were not prespecified.

The prespecified analysis plan of the study consisted of the screening algorithm (Figure 1). We clarified this in the methods section.

**Methods section, pages 5/6**

The first paragraph of your discussion section should summarize the study's findings, and we ask you to expand the text at lines 293-297 to achieve this.

We included the key findings of the study in a novel first paragraph of the discussion.

**Page 16, Lines 384-390**

Please remove trade marks throughout your ms.

We removed the trade marks throughout the document.

Please add a completed checklist for the most appropriate reporting guideline, e.g., STROBE, as a supplementary document, and refer to this early in your methods section. In the checklist, individual items should be referred to by section (e.g., "Methods") and paragraph number rather than by line or page numbers, as the latter generally change in the event of publication.

We believe the STROBE to be the most appropriate reporting guideline for our study and added a checklist as supplementary file.

**Page 5, first paragraph**

*** Reviewer #1:

I confine my remarks to statistical aspects of this paper.  These were quite simple, but appropriately so. However, they were not adequately described in the methods section. The "statistical analysis" section is now 3 lines long. It needs to say what was done and how it was done, even if it's simple. It also needs to describe how variables were operationalized. E.g. what were the "3 routes" to Europe? How were people assigned to one of them? What is "nutritional status" (from the results, it seems like it is BMI ... which isn't really a measure of nutrition, it's a rather poor measure of obesity but a person can be obese and malnourished)/ This sort of thing should be done for all the variables.

We agree that a more detailed definition of variables and more details on the methods section would strengthen the manuscript. We therefore added a paragraph on the definition of variables at the end of the methods section. Furthermore, we gave more details on the statistical analysis, which indeed is not complex in this descriptive study.

**Page 7 (L216) – page 8 (L228)**

More specific comments:

Lines 92-93  These adjectives "limited number" and "select population" apply to all studies including this one. Please give details and say why your paper is better.

We improved the text in order to give the reader a more concise comparison of the mentioned studies with our manuscript.

**Page 5 L132-134**

Lines 163-164  I'm a little confused here. What is "documented date of birth"? How was it determined? What about undocumented DOB - just ask the person their birthday?

In many cases, the URM did not know (or potentially did not want to reveal) their exact date of birth, nor had they official travel documents. In those instances, German authorities assigned them an arbitrary date of birth based on the age information they gave on their identity card, usually January 1^st^ of a given year.

Line 166-167 Certainly 6.0% girls seems low, but how do you know it is "underrepresented"? It's at least theoretically possible that nearly all URMs are boys. Do you have evidence one way oir the other?

We rephrased this sentence to make its meaning more unambiguous.

**Page 8, L239-240**

Line 175 and 178 Please give interquartile range, as well

We included the interquartile range for both the travel time to Germany (IQR 7-18 months) and the time from arrival in Germany to the screening visit (IQR 2-4 days).

**Page 9, L248-251**

Figure 2 D - This isn't really a good graph. Why was this unusual format chosen? Maybe just text about this (it's only 3 pieces of information).

We agree with reviewer 2 that the representation is unconventional and changed Figure 2D to a more classical graph (pie chart).

*** Reviewer #2:

Overall this is an important descriptive paper for a large population of URM who live in Germany and throughout the EU.

Introduction:

- 2nd & 3rd lines of 1st paragraphs would be strengthened by: removing the 2nd line or adding what kinds of challenges: economic, educational, health etc.  For the 3rd line, I would recommend reframing to the fact that infectious diseases have varying endemicities globally rather than framing it as it is reasonable to assume that the endemicity is higher in place where refugees are migrating from.

We are grateful for the suggestions and adapted the manuscript accordingly

-Please more explicitly state the research question/objective

The research objective was defined in the 6th paragraph of the introduction. We agree that

the research goal should be defined as precisely as possible and therefore rephrased the beginning of the paragraph to make it more comprehensible.

**P 5 L143-145**

Methods:

-Please describe the types of patients cared for at a private practice in southwest Germany.

We are grateful for this comment and extended the description of the pediatric practice

**P6 L158-160**

-Figure E. Please revise "mental problems" to say "mental health concerns"

We agree with Reviewer 2 that mental health concerns is a more appropriate description and changed Figure 2 E accordingly. We also changed this term in the methods section.

Results:

Very well described, thorough discussion. Well used tables and figures.

Discussion:
It would be helpful to further describe a brief line or two on how the study is similar or different to Switzerland and SE Germany as described, in demographics, screening or ID prevalence?

We are grateful for this comment and added more information on the cited studies. However, comparative analyses are difficult given the variances in the study designs (retrospective versus prospective design; inpatients versus outpatients).

**Page16 L393- Page 17 L400**

Incorporation of the findings of this paper on Hepatitis B by Yun et al includes a large cohort of youth from E. Africa and would be a fitting for comparison, in addition to their discussion on immunization.
Increasing Hepatitis B Vaccine Prevalence Among Refugee Children Arriving in the United States, 2006-2012.
Yun K, Urban K, Mamo B, Matheson J, Payton C, Scott KC, Song L, Stauffer WM, Stone BL, Young J, Lin H.
Am J Public Health. 2016 Aug;106(8):1460-2. doi: 10.2105/AJPH.2016.303203. Epub 2016 Jun 16.

We are thankful the recommendation to include this important paper. We added the reference to the manuscript together with a short note.

The findings of eosinophilia not being predictive of parasitic disease which supports the rationale has been described in a few studies in North America that would further strengthen the discussion on this topic, and may suggest the need for an alternative approach to parasitic disease screening vs. presumptive treatment among URM in Germany. Potential Papers to consider:
 Eosinophilia: A poor predictor of Strongyloides infection in refugees.
Naidu P, Yanow SK, Kowalewska-Grochowska KT.
Can J Infect Dis Med Microbiol. 2013 Summer;24(2):93-6.
Eosinophilia and the seroprevalence of schistosomiasis and strongyloidiasis in newly arrived pediatric refugees: an examination of Centers for Disease Control and Prevention screening guidelines.
Dawson-Hahn EE, Greenberg SLM, Domachowske JB, Olson BG.
J Pediatr. 2010 Jun;156(6):1016-1018.e1. doi: 10.1016/j.jpeds.2010.02.043. Epub 2010 Apr 18.
High prevalence and presumptive treatment of schistosomiasis and strongyloidiasis among African refugees.
Posey DL, Blackburn BG, Weinberg M, Flagg EW, Ortega L, Wilson M, Secor WE, Sanders-Lewis K, Won K, Maguire JH.
Clin Infect Dis. 2007 Nov 15;45(10):1310-5. Epub 2007 Oct 11.

We are grateful for this constructive comment as it indeed supports our finding of unclear relevance of asymptomatic eosinophilia and included all three references.

The discussion of the strong need to weave together surveillance systems and results was well described in a recent paper about migrants in the UE by Kunst et al and team and would further strengthen the basis for your argument in this area:
Int J Tuberc Lung Dis. 2017 Aug 1;21(8):840-851. doi: 10.5588/ijtld.17.0036.
Tuberculosis and latent tuberculous infection screening of migrants in Europe: comparative analysis of policies, surveillance systems and results.
Kunst H1, Burman M1, Arnesen TM2, Fiebig L3, Hergens MP4, Kalkouni O5, Klinkenberg E6, Orcau À7, Soini H8, Sotgiu G9, Zenner D10, de Vries G11.

We are grateful for this suggestion and added the reference to the text.

*** Reviewer #3:

PLOS Medicine Review PMEDICINE-D-19-03543
Comprehensive infectious disease screening among a cohort of 890 unaccompanied refugee minors in Germany from 2016-2017

Thank you for the opportunity to review the above manuscript. The authors outline infectious diseases screening on a large cohort of predominantly male adolescent unaccompanied refugee minors (URM) presenting in transit to a paediatric practice in South West Germany. Given the lack of symptoms in many URMs, their finding appear to support universal infection screening regardless of clinical symptoms. It should be noted however that the clinicians did not complete full screening when compared to other published international refugee health management guidelines but an abridged version (local German guidelines).

The comments below are intended to improve the clarity of the manuscript and thus the message for the wider readership.

MAJOR COMMENTS
(1) It would be useful to comment upon the structure of the paediatric practice (which undertook screening) and how the infectious diseases screening of the URM was incorporated into the holistic health assessment of the cohort. Specifically, how were the patients managed with respect to treatment of identified diseases/infections and subsequently followed up?

We gave more details on the paediatric practice, as also suggested by reviewer 2.

**P6 L158-160**

(2) The authors state that the German recommendations were not based on local data; the Australian (2009 and revised 2016; Chaves et al) and Canadian (2011 Pottie et al) guidelines are evidence based guidelines with specific details relating to epidemiology of similar cohorts screened (e.g Mutch et al 2012 Journal of Paediatrics and Child Health, Paxton et al 2019 JPCH). Given this fact, I am querying why malaria was not included in the screening protocols, particularly as many URMs appeared to originate from (or transit through) malaria endemic regions. This would also be important to consider in your findings of peripheral eosinophilia and is a limitation of this paper.

Thank you for raising the important consideration of malaria screening. Malaria tropica could be ruled out in all URM due to the length of the travel. Potentially we might have missed some patients with other malaria subforms, we suspect this is limited to a few cases since non of the patients with unexplained eosinophilia appeared clinically ill or reported fever spikes.

**P18 L434-436**

(3) Was broader STI screening considered in this cohort (e.g. HCV, syphilis, chlamydia, gonorrhoea)? This is a high risk/vulnerable population (especially for the small percentage of URM females): other intentional screening recommendations include routine testing of this cohort due to heightened risks related to gender based violence, which are often unreported.

We did not perform broader STI screening in our cohort.

None of the patients reported symptoms suggestive of STI, and patients routinely received a urine dipstick test in addition to a full body exam. In that way, we did not have suspicion for any of the mentioned STIs in our cohort (apart from HBV). However, we agree with reviewer 3 that broader STI screening should be considered in the future, as STI-related symptoms have a high risk of underreporting by the patient.

(4) The authors stated that 'no antiviral treatment was instituted" (Results section HBV and HIV infection line 210) - does this specifically apply only to the cohort that were HBV infected or inclusive of the HIV positive patients? If no treatment was commenced, how was this clinically justified for the HIV cohort? What follow-up, counselling and referrals were put in place?

For HBV, we did not recommend antiviral treatment particularly since we could not prove the chronicity of infection during the initial visit (although being obviously very likely). All three HIV patients obviously started HAART after extensive counseling on the disease (in presence of a professional translator). We apologize for this misleading sentence and corrected this.

**P11 L283-291**

(5) Did the practice undertake review of immunisation status (e.g. rubella, varicella, HBV, HPV)? What catch up immunisation processes were put in place for this cohort? Specifically for the HBV non-immune cohort, how did you commence catch up regimens (in the context of high prevalence of HBV infected URMs in this cohort)?

Catch-up vaccinations are obviously of enormous importance for this high-risk population and were initiated on the day of screening (given contraindications were absent). The vaccination schedule was in accordance with the German recommendations for missed immunisations [Pfeil et al. 2015]:

All URMs received a first dose of MMRV and DTPaIPV vaccines. During the winter months, URMs also received a dose of influenza vaccine at the screening visit. Follow-up vaccinations (two doses of MMRV and three doses of DTPaIPV in total) as well as vaccinations against HBV (three doses), pneumococcus (one dose) and HPV (two to three doses according to age) were deferred to paediatricians/general practitioners who took over medical care of the URMs in the community. We included a paragraph on vaccination in the manuscript.

**Page 6 L179 – Page 7 L188**

(6) It is unclear how nutritional status was ascertained. Was this based on normal haemoglobin and MCV levels on the FBP and normal BMI status? Were iron and vitamin D levels assessed in this cohort? There are data pertaining to children with LTBI who have low vitamin D levels, having an increased risk of progression to active TB. Given that 30% of the cohort with an IGRA were found to have LTBI, this is an important consideration. Were (1) vitamin D levels in this cohort and (2) how did you institute prophylactic or active therapy in this cohort?

This is an exciting question, but we did unfortunately measure Vitamin D levels in our cohort. For clarification, we altered the heading of Figure 2B to BMI (rather than nutritional status).

For the patients diagnosed with active tuberculosis, we initiated standard treatment (isoniazid/ rifampicin/pyrazinamide plus ethambutol based on risk for chemoresistance or patients with complicated TB) after pathogen isolation. We took great efforts to detect TB culturally in the patients in order to treat the patients according to drug-sensitivity testing (as the disease could have been acquired during the escape and resistance profiles could therefore not be estimated). While the diagnostic yield by sputum alone was rather low, we had high success rates using endobronchial ultrasound (EBUS)-guided transbronchial needle aspiration (TBNA). We are currently preparing a separate manuscript on TB diagnostics and therapy in refugee minors (accompanied and unaccompanied) as it would be beyond the scope of this study.

For LTBI, we generally recommended isoniazid prophylaxis for the younger patients (below 17 years), but did not immediately initiate prophylaxis in adolescents >= 17 years especially when we had concerns regarding treatment adherence (and/or therapy safety in case of missed follow up visits for liver toxicity assessment).

We summarized these aspects in the manuscript.

**P14 L354-359**

(7) I note that almost a quarter of the cohort have "mental health" symptoms at first assessment. How were the wider components of refugee health assessment (e.g. education, dental health, immunisation, trauma, malnutrition, haemoglobinopathy screening, non-communicable diseases etc) dealt with in this practice or were they all referred on to other providers?

We are grateful for the comment regarding additional components of refugee health. Patients with suspected mental health concerns were referred to the Department of Child and Adolescent Psychiatry at the Medical Center, University of Freiburg.

While we entirely agree that assessment of education is of enormous relevance for refugees, we did not systematically collect this information as this was not the focus of our study. All refugee minors in Germany are looked after by a staff of social workers, thus education and German language courses are generally offered to the refugee minors. If other, non-communicable health conditions were noted during the screening (eg anemia), appropriate diagnostics was performed at the local practice or the patients were referred to specialist providers. Haemoglobinopathy screening was performed in case of unexplained anemia (i.e. after exclusion of iron deficiency). The important aspect of vaccination was added into the manuscript (see comment above).

**P 16 L379-381**

(8) How do the German guidelines align with the international guidelines (Aust/Canada) which recommend routine screening for all refugee children and adolescents across all of the above domains (not just infectious diseases focused)?

As mentioned in comment (7), social workers were in charge of aspects like language and education. Due to the enormous peak of refugees in Germany 2015-2016, the resources regarding both medical and social care were limited and it was unfortunately not possible to cover all screening items from the mentioned international guidelines. We added a statement to more precisely explain this.

**P 16 L379-381**

(9) What changes to clinical practice guidelines will be (or have been implemented) as a result of these findings? This is an important aspect of this study to include in your discussion, including whether broader screening should be implemented e.g helminth serology, other clinical domains (in keeping with other international processes).

This is a very important point. We provided more information on the ongoing efforts of guideline development of health-care screening for refugee minors in Germany and beyond.

**Page 20, L487-489**

MINOR COMMENTS
(1) Limitations; the lack of malaria testing and serology for schistosomiasis and strongyloidiasis is am important consideration and likely underestimates the helminth load demonstrated in your results. Many refugees are asymptomatic with respect to infection; this would also impact on your assessment of eosinophilia (used as an adjunct to serology).

We are grateful for bringing this important limitation and possible explanation for the unclear eosinophilia in a significant proportion of patients. We included this limitation in the text.

**Page 18, L428-436**

(2) What proportion of the URMs had a protracted refugee transit (> 5 years)? Did this have any bearing on the prevalence of infections (e.g. scabies, malnutrition, dental caries, HBV)?

6 patients of our cohort had a refugee transit exceeding five years, they did not suffer from caries or chronic infections. Unfortunately, this small number does not allow for firm conclusions, so we would prefer not to speculate on this in our manuscript.

(3) Some typographical errors are noted in the paper; particularly in the abstract. It would also be better to use the term "practical" rather than "practicable" for ease of reading.

We have thoroughly proofread and corrected the manuscript.

(5) How did you address language and literacy barriers within the screening process?

All visits were performed with the help of an interpreter, the service was organized by the social workers. At the University Medical Center, interpretation was also possible through a collaborating private company offering live-streaming translation services under the condition that the patient consented this translation service.

**P6 L 164-166**

(6) In your introduction, the authors state that chronic parasitic infections may lead to problems such as anaemia, nutrient deficiency or stunting. What was the correlation in this cohort?

Since we did not perform systematic laboratory measurements of nutritional status, we are not able to perform statistics regarding this question. For anaemia and BMI, there were no statistically significant differences between infested and non-infested subjects. Given the discussed limitations of parasite screening however, it could potentially be possible that parasite-negative patients (as defined by negative stool samples) were indeed infested with Strongyloides spp. or other parasites. Thus, we would feel more comfortable not to include statistics here.

(7) Where there any differences in the infection profiles between female and male URMs?

Since the cohort consisted of 54 female refugees vs. 836 male subjects, it is hard to draw reliable statistical conclusions. More female than male refugees were diagnosed with scabies (26.0% vs. 14.5%) while none were diagnosed with HBV, HIV or pulmonary tuberculosis. Due to unequal sample sizes, we would like to avoid speculations on whether these effects are real, especially since there is no rationale for omitting screening examinations based on gender.

(8) Data analyses (plural) is the correct term; use of analysis incorrectly through the manuscript is noted.

We corrected this error.

(9) Age distribution is skewed (ie non-normally distributed). Median and interquartile range would be more appropriate to use rather than mean age.

We added the interquartile range (15-17 years) and median age (16 years) of the study subjects.

**P8 (L233-234)**

(10) The authors are incorrect in stating that no patients were connected with HCV as it was only tested for in a very small subset (21 patients). This should be corrected and noted in limitations.

Thank you for important concern, we added a comment on this important limitation in the manuscript.

**P17, L421-422**

(11) Looking at BMI status of patients with respect to TB status, it appears non-significant; can the authors confirm this interpretation?

We are grateful for this interesting question. The assumption of Reviewer 3 is correct, the BMI is only slightly different between non-TB patients, LTBI patients and patients with active tuberculosis, which does not reach statistical significance (TB vs. non-TB patients: p=0,6).

Important note: During the analysis, we noted that the BMI values were slightly miscalculated. We corrected the error (which did not affect the outcome and non-significance of the differences). We regret this error and want to apologize for any confusion it may have caused.
