# Supplementary material for: Comprehensive infectious disease screening in a cohort of unaccompanied refugee minors in Germany from 2016 to 2017: A cross-sectional study
Source: PLoS Med. 2020 Mar 31;17(3):e1003076. doi: 10.1371/journal.pmed.1003076 (PMC7108686; doi:10.1371/journal.pmed.1003076)
Supplement: Attachment — Submitted filename: Reply to editors Feb17.docx [file pmed.1003076.s009.docx]

**Comprehensive infectious disease screening in a cohort of unaccompanied refugee minors in Germany from 2016 to 2017: a cross-sectional study**

***PMEDICINE-D-19-03543R1***

**Requests from Editors:**
   
Please remove the word "large" (cohort) from the title; and add a date range for when the study was done.

done

Please also remove "large" from line 35.

done
   
At line 47, please make that "criterion".

done
   
At line 56, please make that "were detected clinically" (assuming this is correct).

done
   
We suggest noting the prevalence of mental health disorders in your abstract.

As stated in the Methods section, a systematical mental health screening was not part of the study. If a patient with mental health concerns was identified in due course, adequate counselling was provided. However, the number of patients we identified as having mental health issues is certain to have been underestimated. For this reason, we prefer not to highlight this information in the abstract.
   
At line 64, please substitute the word "fleeing" with "migrating" or similar.

done
   
At lines 79 and 388, would adapting the wording to "... were not detected clinically." be appropriate?

done
   
At line 83, please remove the word "completely".

done
   
Please adapt the sentence at line 115 to make it clear that the prevalence is lower in Germany (e.g., "In Germany ... a twenty-fold lower prevalence.").

done
   
At line 162, please make that "non-prespecified".

done
   
At line 169, please substitute "transit" (or similar) for "flight".

done
   
At lines 170 and 173, please substitute "drug [substance] use" (or similar) for "drug [substance] abuse".

done
   
Please reword the text at line 209 to make it clear whether the legal guardian or municipality provided consent. Please make it clear whether consent was informed, and how it was established (written/verbal). The wording in the text seems to contradict the note in your response to editorial points that it was "not possible to prospectively obtain informed consent" and we ask you to make the actual situation clear in the revised ms.

Consent was provided by the legal guardian who was assigned by the municipality prior to the URM’s health care screening. In addition, the URM was informed by an interpreter present at the health check about the planned procedures and the URM was asked for verbal consent. We did not require written consent from the URM.

At line 213, please make that "Data analysis was performed in an anonymized fashion." (assuming this is correct).

done
   
At line 234, we suggest making the information less specific, e.g. "... the youngest ... was 9 years of age".

This sentence was removed.
   
 At line 268, can you be more specific about "mental health issues", i.e., were disorders noted?

done
   
At line 282, should that be "anti-HBV immunity"?

No, HAV is correct (hepatitis A). We modified the sentence for better clarity.

At line 387, please amend the text to "... were diagnosed in a small minority of refugees" or similar.

done
   
At line 388, please make that "were not recognized clinically".

done
   
At line 389, please adapt the text to "because they showed" or similar.

done
   
At line 399, please substitute "endure an escape" with "underwent a journey" or similar, and remove the word "flight".

done
   
At line 413, please avoid the word "subjects" (in favour of "people" or similar). Would "provided" be a good substitute for "vigorously controlled"?

done
   
 At line 420, please substitute "countries" or similar for COI.

We removed the abbreviation COI from the text and instead referred to country of origin.
   
 At line 435, please expand on "M.tropica", as this does not appear to occur earlier in your ms.

done
   
 At line 452, we suggest revisiting the text "reconfirms the minimal risk". It may be that noting the small proportion of sputum positive patients could suggest a low risk of onward transmission. You might wish to briefly mention local policies on BCG vaccination, which could be relevant to this point.

As we are not aware of studies showing effectiveness of BCG vaccination in preventing onward transmission of TB, we did not specifically comment on this issue in the text. We did add the word “onward” to describe onward transmission.
   
 At line 462, would that be "... reached 23.3% in this study"?

done
   
At line 483, please substitute "migrating" for "immigrating".

done
   
At line 485, should reference citations be substituted for "(Pottie; CDC; Chaves)"?

done
   
In reference 20, please abbreviate "World Health Organization" as "WHO", or not at all.

done
   
Please revisit reference 31, as the title appears to be partially duplicated.

done
   
In figure 2, please remove the word "escape" (route to Europe).

Done

**Comments from Reviewers:**
   
*** Reviewer #1:
   
If the editors want to publish as is, that's OK, but I'd rather delete the pie chart and just describe the data (it's only 3 points of information). Peter Flom

We would prefer not to change the graphic and will leave the final decision to the editorial team.

*** Reviewer #2: 
   
-Reviewer 1 asked a question about the documented date of birth, and it was answered in a way as to clarify this question. The authors response should be added to the paper for clarity for the reader.

We reviewed the explanation within the text. To us, it seemed sufficient for comprehension. For this reason, we did not expand upon the explanation, as we felt that it would not be decisive for readers.

-Reviewer 2 recommended adding additional citations to the text. It's noted that these citations were added, however, additional sentences were not added placing this new study in the context of these previous studies. That component is very important. Additional comparison and potential emphasis or disagreement with these prior papers is needed to place this new study in context.

We have added information about other studies based upon the reviewers’ suggestions.

-Reviewer 3 queried about STI testing. Given this study is focusing on ID it is important that this discussion and additional commentary from the authors is included in the discussion of this topic. Many unaccompanied refugee minors have experienced trafficking and are at high risk for it. Like many adolescents, they may not reveal their sexual history particularly for non-consensual sex, or that differs from their definition of sex. Arguably they are at high risk and more discussion of STI testing should be included in the discussion as well as acknowledged in limitations.

We have added a note to clarify that the current study did not address the screening of STDs except for HIV and HBV.
   
-Reviewer 3 raised a very important concern about language and literacy barriers. In the body of the text the more detailed description of the interpretation should be added as well as the use of interpreter rather than translator.

We have changed and replaced the word “translator” with the word “interpreter”. As described in the Methods section, an interpreter was always present at the interview — either in person or else via video service.
   
-Reviewer 3 raises an excellent point about chronic parasitic infection contributing anemia, nutrient deficiency or stunting being included in the introduction this sentence would benefit from a citation. Further, the description provided by the authors should be included in the paper. Please change infest to infect, throughout this comment and the paper.

We have replaced the word “infestation” with infection throughout the text.
   
-Reviewer 3 raises an important point about the data as it relates to the few female refugees in the study. Please add your response to the comment to the limitations section.

done
   
Introduction:
-For line 99, "...it seems reasonable to screen refugees for ID that bay me more prevalent in this population." Please provide more context for this assertion. I imagine that the authors mean that ID is higher prevalence in low and middle income countries and more refugees originate from these countries, however, I think this additional context is needed.

done
   
-In the conclusion to paragraph 1, I think it is important to also include that limited/no access to care may also contribute to delays in diagnosis

done
   
-Paragraph 2: It would be helpful to provide some context as to why Europe and Africa as continents are being compared specifically. I imagine this is due to the high number of URM from Africa. Further, please add language to reflect that these difference in prevalence relate to being low and high resource places rather than something inherent to the continents.

After reviewing the text as a whole, we felt that readers would be able to deduce this from context. For this reason, we did not add further comments.
   
 Methods:
 -It is not clear what language the survey was written or administered in and if it was interviewer/interpreter assisted in completing this. were the questions developed with language and culturally concordat team members? Please provide additional context here.

The questionnaire was provided in German and was used by the screening physician. As previously described, communication with the patient (URM) was done with the assistance of an interpreter.
   
 Results:
 -Please remove line 234-235 about the youngest study team member as this may be identifying.

done
   
-Figure 2: There are errors in the key as I believe C and E are flipped. Additionally it may be fitting to call it systems of clinical signs and symptoms, or systems and diagnoses given it relates to body systems and then scabies

We have corrected the legend.
   
-For Line 276-277, the majority should be placed in the context of the fact that it was only patients among the 24 that had additional testing. This would not reflect the majority overall, and is therefore misleading.

done

-Table 1, please move the section related to the 24 of 60 patients to another table or to another column as it is important that the % are interpreted to be reflective of the 24 patients not the overall 776 that had testing for HBV

We have deleted those numbers in Table 1.
   
-Line 341 please remove the frame "surprisingly" high, since this is the results section it should not include such descriptive words and opinions of the authors.  Similarly please remove interestingly.

done
   
-Line 343 Please remove the line that TB patients were not contagious, or elaborate on the context. Clinicians commonly say that anyone with active disease is considered contagious.

done
   
-Line 346-347: Please remove the idea that there was a tendency toward lower BMI in TB patient as p=0.6 is far from significant.

done

-Paragraph 375-381 may fit best in the methods section as it does not seem like it follows from the results as it is not about something that was screened for.

done
   
Discussion:

-Line 399-400 please add the citation that the finding is in reference to.

This is our own finding. We have edited the sentence for clarity.
   
-Line 403-404 please provide additional context as to why it si worth noting that the URM are predominantly of African origin. Is it similar or different from prior studies?

done
   
-Line 410-413: Please revise these lines or remove them as it is inaccurate as currently framed. The viral load being high in 20% of patients is 5 patients given that there were only 24 patients who had their viral loads evaluated. This is 0.6% of the population of those tested for HBV.  Additionally, it would strengthen the discussion to add that HBV may be acquired through vertical transmission, blood or sex. It would be helpful to provide some additional context around what other forms of prevention might be helpful to consider as compared to large scale prevention in the country of origin.

We have removed the “20%” and also have removed this information from Table 1.
-Line 455-456 please include a citation for the recommendation around LTBI vs. active TB disase screening

done

-I recommend removing Table 4, the sample size is <5 in the majority of the cells (making it difficult to interpret), and individuals with latent TB do not have symptoms suggesting that those that have cough, weight loss or fever have another illness.

We concur that this data has low power due to the limited number of cases observed. Accordingly, we have moved Table 4 to the Supplements section.
   

Production issues as of February 14 2020 CC: [plosmedicine@plos.org](mailto:plosmedicine@plos.org)

Dear Dr. Elling,

Thank you for resubmitting your manuscript entitled "Comprehensive infectious disease screening in a cohort of unaccompanied refugee minors in Germany from 2016 to 2017: a cross-sectional study" to PLOS Medicine.

At this stage, we need to make sure that certain production issues are addressed in order to ensure a smooth handover to our production team, should the manuscript be formally accepted.

Can you please address the following issues as soon as possible? Your paper has been sent back to enable you to make these changes.

FIGURES and GRAPHICS:

1. Please ensure that all main figure files are cited in ascending numerical order in the main text of the article.

corrected

2. Please ensure that all main figures are referenced in the manuscript as Fig 1, Fig 2, etc. (including capitalization), rather than Figure 1, Figure 2, etc. Please note, however, that the file names themselves must not include the space, i.e. Fig1.tif, Fig2.tif, etc.

corrected

3. Please ensure that all main figure legends are placed after the paragraph containing their first in-text citation.

corrected

4. Please include the location from which you obtained the base map used in Fig 2 in the legends for this figure. Please note that, because all PLOS articles are published under a CC BY license (creativecommons.org/licenses/by/4.0/), we cannot publish proprietary maps such as Google Maps, Mapquest or other copyrighted maps. If your map was obtained from a copyrighted source please amend the figure so that the base map used is from an openly available source.

The figure is was generated using R and is therefore open source. We clarified this in the methods section:

“Statistical analyses were performed using R (v 3.4.4) and the dplyr (v 0.7.8) package. Graphics were generated using the open source R packages ggplot2 (v 3.1.0) and maps (v 3.3.0) for the map in Fig 2C, as well as Adobe Illustrator CS6, Microsoft Excel and GraphPad Prism (Version 8). Comparison across patient subgroups was carried out using two sample t test for continuous variables and Chi square tests for categorical variables. P values <0.05 were considered significant.”

7. Figure 1 has been modified to meet with formatting requirements. Please review the revised figure to ensure that it meets with your approval. Please note that if you approve of this revised figure, you do not need to upload it to the system.

-Figure 1 was changed to RGB color mode. Ready for publication with author approval.

We are approving the change to RGB mode.

-Please note that Figure 2 does not currently comply with our publication requirements. For further instructions to assist with your Figure revisions, please refer to our Figure Guidelines at: <http://journals.plos.org/plosmedicine/s/figures>.

If you need assistance, please contact us directly at [graphics@jjeditorial.com](mailto:graphics@jjeditorial.com) so we can further help with this matter. Please send any relevant source files and tell us what application(s) and what operating system you are using.

-Rename Figure 2 to match PLOS formatting e.g. Fig(number).(file extension) ex. Fig2.esp

corrected

-Figure 2 exceeds the dimension limits for figures. Figures must be less than 19.05 cm in width, and less than 22.225cm in height (excluding white space around your artwork). Please make the necessary adjustments and re-upload your figure(s).

corrected

-Figure 2 has been submitted in Bitmap CMYK, a color space which we do not accept for Figure files. Please provide us with Figures in RGB (millions of colors), 8 bits/channel, saved as a high resolution TIFF or EPS.

corrected

-Figure 2 currently exceeds the maximum acceptable file size for figures (10 MB). Please modify your figure accordingly so that the file size is 10 MB or lower. If you are unable to resolve this issue as an EPS, please save this Figure as a TIFF (with LZW compression; [http://journals.plos.org/plos[x]/s/figures](http://journals.plos.org/plos%5Bx%5D/s/figures)) in order to reduce the file size.

corrected

TABLES and BOXES:

1. Please ensure that all main tables in their entirety (including titles and footnotes), are placed after the paragraph containing their first in-text citation.

corrected

2. Tables 1 and 2 use returns to control text wrapping. This is indicated by a [¶ or ↵] symbol when "Show Paragraph Marks" is turned on in Word. Please either split those cells into separate cells to achieve alignment or remove the returns.

corrected

SUPPORTING INFORMATION:

1. Please ensure that each supporting information file has a legend at the end of the manuscript file, after the Reference list.

corrected

2. Please note that your submission cannot contain any references to generic "supporting information" files and instead must refer to specific files using our naming convention (S1 Fig, S1 Table, S1 Text, S1 Data, SI STROBE Checklist, etc.)

corrected

3. Please ensure that all Supporting Information files use the following format verbatim throughout the manuscript (including legends and all in-text citations): S1 Fig, S1 Table, S1 Text, S1 Data, etc. Each file type should start at 1 and increase numerically. Please note, however, that the file names themselves must use an underscore rather than a space, i.e. S1_Fig.tif, S1_Table.xlsx, S1_Text.doc, S1_Data.xlsx, S1_PRISMA_Checklist.docx, etc.

corrected

If you have any questions please feel free to contact us at [plosmedicine@plos.org](mailto:plosmedicine@plos.org).

Best regards,

Jessica Hyde, Publications Assistant, PLOS Medicine

[plosmedicine@plos.org](mailto:plosmedicine@plos.org)
